# The perceived self-efficacy of senior, middle, and operations managers of the incident command system dealing with emergencies and disasters during the COVID-19 pandemic

**DOI:** 10.1186/s12873-023-00904-9

**Published:** 2023-11-09

**Authors:** Asiye Aminafshar, Majid Sartipi, Abdolrazzagh Pakzad

**Affiliations:** 1 Health in Disasters and Emergencies Research Center, Institute for Futures Studies in Health, Kerman University of Medical Sciences, Kerman, Iran; 2 Health Promotion Research Center, Zahedan University of Medical Sciences, Zahedan, Iran; 3 Department of Biostatistics and Epidemiology, School of Health, Zahedan University of Medical Sciences, Zahedan, Iran; 4 Health Safety and Environmental Management (HSE), Zahedan University of Medical Sciences, Zahedan, Iran

**Keywords:** Incident Command System (ICS), COVID-19, Perceived self-efficacy, Disasters, Emergencies

## Abstract

**Background:**

Natural disasters, health, terrorism, infectious diseases, and social unrest affect more than 200 million people worldwide each year. The present study is an attempt to evaluate the self-efficacy of senior, middle, and operational managers of the Incident Command System (ICS) of Zahedan University of Medical Sciences in Iran.

**Methods:**

The study examined the perceived self-efficacy of 103 senior, middle, and operational managers of the Incidence Command System (ICS) of Zahedan University of Medical Sciences in 2021. Sampling was done by census using a designed questionnaire based on Bandura’s self-efficacy concepts. Based on Factor Analysis, 4 factors were extracted. The factors were labeled and analyzed.

**Results:**

The number of people who had passed the crisis management course was 57. Seventy-one participants reported their participation in crisis management. The score obtained by men in Factor 3 (F3) was significantly higher than women, but not in other factors. People with stable employment scored far higher in Factor 1 (F1) than those with unsustainable employment conditions. Those who had passed the Crisis Management courses had a higher average score, but only in the three factors 1, 2, and 4, this difference was significant.

**Conclusion:**

Even training the temporary staff is an organizational investment that can return benefits to the system. This enhances their perceived self-efficacy and promotes their commitment to the organization. Therefore, empowering these managers should be a priority.

## Introduction

Natural disasters, health-related problems, terrorist attacks, infectious diseases, and social unrest all around the world affect more than 200 million people annually. These disasters are increasing all over the world [1–3]. Iran is affected each year by an average of 253 risks leading to disasters of various sizes depending on their frequency, extent, and population congestion. Occasionally there may be problems with restoration [4, 5]. Events of moderate severity can also affect the national health and treatment system [6, 7]. The healthcare system plays an effective role in reducing the casualty and death toll in times of crisis. One goal that health care and treatment systems are trying to achieve in responding to incidents and disasters is to reduce and prevent deaths and physical and mental health problems. This aim can never be achieved without proper planning, preparation, and training of the workforce involved in the crisis [6, 8, 9]. Meanwhile, crisis managers are required to provide an access to organized, integrated, easily accessible, and coordinated medical services. Poor planning, inadequate resource allocation, and lack of coordination between different departments make it difficult to provide medical and healthcare services in the event of a disaster [10, 11].

After WHO announced the COVID-19 pandemic, Iran also got involved with the pandemic on February 19^th^, 2020. As of September 2021, more than 54 million infected people were diagnosed and over 117,000 deaths were reported in Iran [12, 13]. From the beginning of the pandemic to August 2021, the incidence of coronavirus infections in Iran was higher than the global average, and the epidemic was in four zones, including Southeast Asia, the Eastern Mediterranean, North Africa, the Western Pacific, and Africa [14]. Based on the data released in the database of Zahedan University of Medical Sciences, Iran, since the beginning of the Covid-19 pandemic, Sistan and Baluchestan province in Iran experienced three peaks of illness in July 2020, November 2020, and July 2021.

Disasters are events that are likely to result in massive complications, mortality, and morbidity in addition to infrastructure damage [15]. WHO defines a disaster as a seriously dissociated performance of a society, resulting in widespread human, economic and environmental losses and negative effects, so its management is beyond the power of the affected society if it just relies on its internal resources. In disaster management, organizations encounter sudden and unexpected changes and problems over which they do not have proper control [16–18]. According to the published reports, 1.6 million people around the world have lost their lives due to natural disasters since 1990, accounting for about 65000 annual deaths [19].

Disasters present many challenges to managers due to their frequency and damage. Therefore, as crisis managers are under maximum stress, they need to develop special skills to make appropriate decisions in the shortest possible time so as to control disasters and reduce subsequent complications on the one hand and lead command teams on the other [17, 20]. Effective management has a paramount role in controlling crises, and coordinating and enhancing the efficacy of actions [21, 22]. Taking skillful and proper measures in difficult and unpredictable situations is the consequence of manager qualification and competency. perceived self-efficacy as a component of managers’ qualification and competency involves one’s belief in their own capabilities to achieve certain goals [23]. For Bandura, it reflects one’s beliefs in their capabilities to exercise control over their functioning and over events that affect their life. In fact, the perceived self-efficacy in completing a task reflects a person’s self-confidence and desire for a particular behavior [24, 25]. Relying on personal beliefs in their abilities, managers can predict future performance in dealing with disasters and crises [21, 26].

People with higher self-efficacy believe that they can have proper job performance in stressful occupational situations. They are more inclined to take precautions against problems ahead. But people with lower self-efficacy prefer avoidance strategies. Higher self-efficacy affects how people respond to work-related stressors [27]. Self-efficacy influences the outcomes because it involves one’s self-confidence in controlling thoughts, emotions, and actions. Managers’ self-efficacy in such issues as situational decision-making, evidence-based judgment, prioritization, planning, stress control, and effective communication plays an important role in crisis management because the first step in overcoming a crisis is to feel that you are competent enough to respond to it. Therefore, the present study aimed to investigate the perceived self-efficacy of senior, middle, and operations managers of the Incident Command System (ICS) dealing with events and disasters during the covid-19 Pandemic, in a major state university in Iran (i.e. Zahedan University of Medical Sciences) as a major responding organization against disasters and incidents during the pandemic.

Perceived self-efficacy is affected by vicarious experience, previous mastery experiences, verbal persuasion, and physiological and emotional responses. Previous successful experiences as the most important factor in reinforcing the perceived self-efficacy play a paramount role in facing similar situations [24]. Yarmohammadian et al. (2013) evaluated the relationship between self-efficacy and competency of some managers during the crisis in Isfahan, Iran. They observed the managers’ desirable self-efficacy in communicating with team members and announcing the emergency conditions. However, their self-efficacy was not desirable when it came to coordinating between team members and allocating resources before the crisis, and reducing staff stress during the crisis. They found a significant positive correlation between the managers’ competency and self-efficacy [21]. Kafi et al. (2016) also examined the relationship between hardiness, resilience, and self-efficacy among the crisis managers of the Red Crescent Society in a number of provinces in Iran. They found that by believing in their abilities, managers can shape their cognition, attitude, behavior, and performance to maximize their potential and increase resilience in difficult times [28]. In a meta-analysis, Stajkovic and Luthans (1998) found a strong positive relationship between self-efficacy and job performance; they also observed that occupational complexity would tend to undermine such a relationship [29]. Also, Baas et al. (2003) studied the self-efficacy of nurses caring for patients with heart failure and found that implementing educational programs was effective in enhancing the self-efficacy and performance of nurses [30]. Jaafaripooyan et al. (2017) assessed the self-efficacy of senior hospital managers in Tehran, Iran, and found that managers had a high level of self-efficacy in facing crises. The perceived self-efficacy of male participants was higher than that of females. In addition, married managers showed better self-efficacy than single managers. Self-efficacy scores were also higher among managers with previous crisis management experience [31]. Ventura et al. (2015) examined the role of self-efficacy as a predictor of job burnout, and confirmed that higher levels of perceived self-efficacy encouraged managers to take part in challenges; also, these managers had much fewer hindrance demands in achieving their goals [32]. Wolfrum (2020) analyzed the self-efficacy of crisis leaders in the United States, and reported that the occurrence of a crisis entailed effective management abilities; also there was a positive correlation between managers’ effective leadership and self-efficacy during the crisis. However, there was no significant association between managerial self-efficacy and demographic characteristics such as education level, previous experience, and involvement in crisis management [33].

## Methods

This cross-sectional and analytical study was conducted to investigate the perceived self-efficacy of 103 senior, middle, and operations managers at Zahedan University of Medical Sciences, Iran in 2020–2021 in dealing with the Covid-19 disease crisis. The study population included EOC (Emergency Operations Center) senior managers, middle managers, and operations managers of in Zahedan, Iran. Overall, 140 people were predicted to participate in the study.

The senior managers, including the commanders and seniors of the university's EOC (finance and support department, planning department, medicine and equipment department, health operations department, and treatment operations department) were included in the study based on the National Response Framework (NRF).

The middle managers include the commanders and seniors of the EOC of health and treatment networks and HICS of hospitals in the covered districts based on NRF, who were included in the study as middle managers.

Operational managers include other EOC members of the district health network and HICS of hospitals who are directly involved in operations and were included in the study as operational managers.

Through a census sampling method, all Incident Command System (ICS) managers at the three levels of managers were invited to participate; however, only 103 participants completed the questionnaire.

**Fig. 1:**
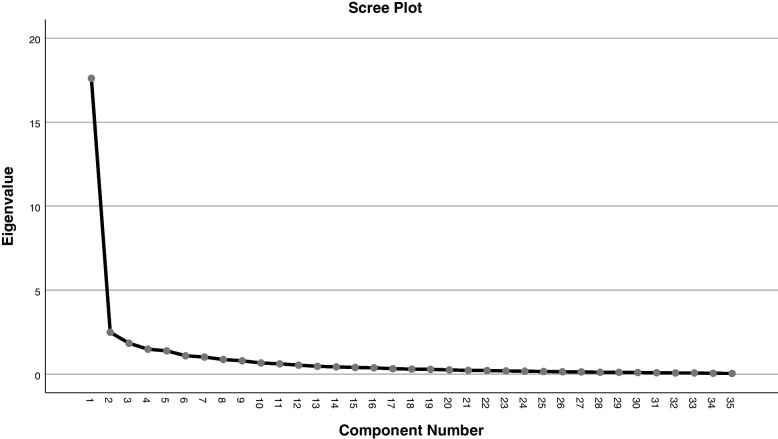
Scree plot of the component number and eigenvalues of questions

We used the Persian version of Bandura's General Self-Efficacy Scale, the validity and reliability of which were already confirmed in a study by Jaafaripooyan [34]. However, since a number of items about managers’ awareness of NRF were added to the questionnaire, the newly-developed scale was also validated by the judgment of an expert panel of psychologists and specialists in health education and disaster health; its reliability was confirmed by Cronbach’s alpha (α = 0.89).z The questionnaire consisted of two parts: demographic information (15 items) and main body (35 items) including NRF-related duties, commandments, coordination and communication, planning, problem-solving skills, and stress and emotional control. Each statement was rated against a 5-point Likert scale ranging from “very high” to “very low”). Since there were too many items and the direct application analysis of each item could not be performed, it was necessary to extract hidden variables from the data. Therefore, factor analysis was performed to reduce the dimensions of the data. First, the feasibility of factor analysis was checked using the Kaiser–Meyer–Olkin (KMO) measure of sampling adequacy and Bartlett’s tests; KMO was greater than 0.5 (KMO = 0.891) [35]; and Bartlett’s test of sphericity equaled 3335.917 (df = 3335.917, *p* < 0.001); it was revealed that correlation between the items was great enough to allow factor analysis. Therefore, the study data was found appropriate for factor analysis. The total variance was explained based on the screen plot where factor #4 showed the curve point, and the remaining four factors (with a total variance %66.267) accounted for the variances of all items (Fig. 1, Table 1).

**Table 1:** Components derived from factor analysis

Component	Number of Questions	Rotation Sums of Squared Loadings	% of Variance	Cumulative %
Factor 1 (F1): Perceived Self-efficacy in the operations commandment	15	7.975	22.785	**22.785**
Factor 2 (F2): Perceived Self-efficacy in planning and improving the technical performance of the operations teams	9	5.931	16.945	**39.731**
Factor 3 (F3): Perceived Self-efficacy in emotional control, coordination, and management	7	5.535	15.815	**55.546**
Factor 4 (F4): Perceived Self-efficacy in familiarity with the crisis management structure and tasks	4	3.753	10.721	**66.267**

In the first phase of factor analysis without rotation, most items were loaded into F1. However, Varimax rotation and re-analysis resolved the problem; 15 items with a loading higher than 0.46 loaded onto F1, 9 items with a loading higher than 0.48 loaded onto F2, 7 items with a loading higher than 0.52 loaded onto F3, and 4 items with the loading higher than 0.7 loaded onto F4 (Table 2). Based on the items and consulting professionals, the factors were labeled as follows: Factor 1 (F1): Perceived Self-efficacy in the operations commandment; Factor 2 (F2): Perceived Self-efficacy in planning and improving the technical performance of the operations teams; Factor 3 (F3): Perceived Self-efficacy in emotional control, coordination, and management; Factor 4 (F4): Perceived Self-efficacy in familiarity with the crisis management structure and tasks.

**Table 2:** Description of factors extracted from factor analysis

	**Factor 1**	**Factor 2**	**Factor 3**	**Factor 4**
**Mean**	57.61	33.04	26.67	13.11
**Median**	59.00	34.00	27.00	13.00
**Std. Deviation**	9.78	6.58	5.01	3.65
**Minimum**	30.00	14.00	14.00	4.00
**Maximum**	75.00	45.00	35.00	20.00

Based on the factor analysis with 35 Varimax rotations, four extracted factors included F1 accounting for 22.785% of the total variances of the items; F2 accounting for 16.945%, F3 accounting for 15.815%, and F4 accounting for 10.721% of all variances. The four extracted factors together accounted for 66.267% of the total variances (Table 1).

Regarding the participants’ responses on a Likert scale (5 for “very much” and 1 for “very little”), the range of minimum and maximum scores for each factor were as follows: 15 to 75 (F1), 9 to 45 (F2), 7 to 35 (F3) and 4 to 20 (F4), respectively. Participants’ mean, median, standard deviation, maximum, and minimum scores for each factor are summarized in Table 2.

## Results

There were 53 male (51.5%) and 50 female (48.5%) participants who were senior, middle, and operations crisis managers. Ninety-six participants (93.2%) were married and the rest were single. In terms of educational level, most (47.6%) had a bachelor's degree. Thirty-four participants (33%) had Master’s and 10 participants (9.7%) had Ph.D. degrees. As for their majors, 47 participants (45.6%) graduated from a clinical major, 36 participants (35%) from a health department, and 20 participants (19.4%) from humanities. Most of the participants (*n* = 75, 72.8%) were officially and permanently employed. Eighteen participants (17.5%) were recruited through temporary contracts. Five participants were hired on an annual contract (4.9%), and another five were on temporary outsourcing contracts (4.9%). They were categorized into 47 therapeutic staff (45.6%), 38 health staff (36.9%), 13 administrative finance staff (12.6%), and 5 blue-collar service providers (4.9%). Of all, 57 participants (55.3%) had already attended in-service crisis management courses. Also, 71 participants (68.9%) reported their active participation at least in one real case of crisis management; they had either experienced or managed between 1 and 10 cases of crisis management (mean = 1.36, SD = 1.84). The most frequent managerial roles included 23 health duties (22.3%), 19 therapeutic operations (18.4%), and 15 logistic administrative roles (14.6%) (Tables 3 and 4).

**Table 3:** Demographic status

	**Number**	**Percent**
**Age (year)**		
< = 30	7	6.8
31–40	38	36.9
41–50	35	34
51 = <	23	22.3
**Sex**		
Male	53	51.4
Female	50	48.6
Sex ratio	1.06	
**Marital status**		
Married	96	93.2
Single	7	6.8
**Education level**		
Bachelor's degree	59	47.6
Master degree	34	33.0
PhD	10	9.7
Other	10	9.7
**Employment status**		
Officially and permanently employed	75	72.8
Temporary contracts	18	17.5
Annual contract	5	4.9
Temporary outsourcing contracts	5	4.9

**Table 4:** Membership in the disaster management system

Title of membership in the Disaster management system	Frequency	Percent
Commander	9	8.7
Senior Coordinator	10	9.7
Senior Safety	6	5.8
Senior Security	5	4.9
Senior Communications	4	3.9
Office Support	15	14.6
Medicine and equipment	4	3.9
Planning	8	7.8
Health operations	23	22.3
Treatment operations	19	18.4
**Total**	**103**	**100.0**

Analysis of variance revealed no significant differences between the four factors extracted from factor analysis and variables such as participants’ organizational status, their occupational classification (health, treatment, and logistics), their role in crisis management, the organizational hierarchy (university, health network, and hospital levels), marital status, educational level and major (*p* = 0.05). Men’s F3 scores (27.7) were significantly higher than women’s F3 scores (25.5) (*p* = 0.028). However, differences were not significant with other variables. Participants with permanent employment status scored higher (mean = 58.3) in F1 than those with unstable temporary employment status (mean = 44) (*p* = 0.001). However, there was no further significant difference between the two groups in the other three factors.

Of all, 57 participants (55.3%) had participated in 1–10 crisis management courses. Participants who had attended crisis management courses showed higher mean scores in all four factors; however, the differences were significant only for F1 (*p* = 0.004), F2 (*p* = 0.005), and F4 (*p* < 0.001). Also, the more courses the participants attended, the higher their scores on F1 (*p* = 0.005), F2 (*p* = 0.003), and F4 (*p* = 0.006) were.

Participation in previous crisis management experiences resulted in higher scores on all four factors compared to participants who did not have such experiences; however, the differences were significant only for F1 (*p* = 0.002) and F3 (*p* = 0.003). The linear regression analysis showed a significant relationship between participants’ employment history and F1 and F3 (Table 5). There was no significant association between employment history and F2 and F4.

**Table 5:** Linear regression for factors and history of employment (Year)

Dependent Variables (Factors)	Independent Variables	Std. Error	Beta	Sig	95.0% Confidence Interval for B	
					Lower Bound	Upper Bound
F1	(Constant)	51.535		<.001	47.187	55.882
	History of employment (Year)	.338	.293	.003	.120	.556
F1	(Constant)	1.134		<.001	21.702	26.200
	History of employment (Year)	.057	.256	.009	.038	.264

Linear regression analysis revealed a significant relationship between F1 and two other factors (i.e. F2 and F3). In addition, F2 showed a significant relationship with F1, F3, and F4. However, F3 was significantly related to F1 and F2; and F4 was significantly associated with F2 only (Table 6). No significant relationship was found with other states.

**Table 6:** Linear regression for factors

Dependent Factors	Independent Factors	β	SE	*P*. Value
F1	F2	0.438	0.116	< 0.001
	F3	0.463	0.152	< 0.001
F2	F1	0.422	0.066	< 0.001
	F3	0.262	0.121	0.005
	F4	0.26	0.125	< 0.001
F3	F1	0.563	0.049	< 0.001
	F2	0.27	0.072	0.005
F4	F2	0.599	0.044	< 0.001

## Discussion

Organizational planning and workforce training positively correlate with the power of medical systems to manage and respond to disasters and events. In fact, organizations with highly trained workforce act more effectively by making prompt decisions, managing stress, and preventing spontaneous disruption; they also have a higher level of self-efficacy [36–39]. Perceived self-efficacy is influenced by vicarious experience or seeing people like you successfully achieve task demands, performance accomplishments, experiences of mastery, verbal persuasion as well as emotional and physiological states. As the most important reinforcement source of perceived self-efficacy, past experiences of success highly influence perceived self-efficacy in similar situations [25]. In this survey, 103 senior, middle, and operations managers (out of 140 managers) voluntarily completed the study questionnaire; they were employed in the health centers, hospitals, and crisis management headquarters of Zahedan University of Medical Sciences, Iran. It was found that 46 of the ICS managers (44.7%) had not attended any crisis management courses. In addition, 25 (35.2%) of managers with crisis management experience reported that they had never attended a crisis management course. The educational qualifications of 49 managers (47.6%) ranged from master’s degrees to medical subspecialties.

There was a significant difference in the gender effect of the senior, middle, and operations managers of the Incident Command System. Almost half of the Incident Command System (ICS) managers (48.5%) were women, reflecting an appropriate gender representation. Chi-square and Fisher’s exact test showed that 88.9% of senior managers, 66.7% of middle managers, and 46.6% of operations managers were men, reflecting a significant difference (*p* = 0.043). The difference means that women are given fewer opportunities to function as senior and middle managers in the Incident Control System, while more are hired as operations managers. While women have many skills and abilities, they perceive less self-efficacy because of ignorance of their own abilities and being ignored by the management system [40]. Senior managers’ confidence in women’s abilities and providing them with equal opportunities to advance to leadership and management positions can pave the way for actualizing their managerial powers and, consequently, enhance their perception of self-efficacy. A survey of governmental hospitals in Tehran, Iran, found that 59% of the participants were women [34], which is in line with the present findings.

In the present study, there was a significant difference between gender and F3. Male participants’ mean scores (27.7) were higher than that of female participants (23.6) (*p* = 0.028). In Wutjatmiko and colleagues’ study, while 75.5% of the participants were women, 56.2% of them perceived low self-efficacy in response to disasters [41], which is consistent with the present study findings.

There was no significant relationship between managers’ employment history and the number of crisis management courses they had taken (*p* = 0.766). The lack of a relationship between managers’ employment history and attending crisis management courses can be attributed to the fact that, first, crisis management courses are quite new and, second, Iran’s Health System has introduced a national disaster response program to medical universities since 2016. Of all the 103 participants, 44.7% had attended no courses in crisis management. Also, 68.9% of these managers reported their management of or participation in a case of crisis control in the past, while 25 of them (35.2%) had not attended any crisis management courses.

There was no significant difference between the four domains of self-efficacy and participants’ educational level; this finding was not consistent with that of Wutjatmiko et al. [41], and Melnikov et al. [42]. Wutjatmiko et al. found that participants with higher educational levels exhibited more information about incidents and disaster management; consequently, they were able to make precise and timely decisions in the event of a crisis [41]. One of the reasons for this difference may be that participants are not trained in crisis management. Also, disaster management courses are not included in the curriculum of the relevant university major.

The results showed that as the number of training courses increased, the values of F1 (*p* = 0.005), F2 (*p* = 0.003), and F4 (*p* = 0.006) significantly increased; this might be attributed to the goal-oriented nature of the courses. However, there was no significant relationship between the F3 score and these courses (*p* = 0.233). Jonson et al. showed that the lack of training courses lowered the participants’ self-efficacy scores. Training and practice are two ways of enhancing one’s perceived self-efficacy in developing skills and tasks required for crisis management [43]. Crisis management courses can promote the participants’ perceived self-efficacy for effective management, leadership, and teamwork [44].

Providing effective training can improve self-efficacy in communications, teamwork, and leadership [44, 45]. Regarding the structure, organizational hierarchy and powerful position of senior managers, their planning and recommendation of training courses for crisis managers will bear more considerable effects. However, in the organizational structure of medical universities supervised by the Iranian Ministry of Health and Treatment, the Incident Control System (ICS) and the pre-hospital Emergency Medical Services are controlled by the Treatment Deputy. Also, the district health networks, the managers of which constitute the incidents commandment system in the periphery, are managed by both the Health Deputy and the Treatment Deputy. The Incident Control System and the pre-hospital Emergency Medical Services are responsible for planning programs for responding to disasters and training in disaster management at universities. At the organizational hierarchy, they are at the same level as the district health networks. Therefore, they lack the authority to seek the participation of managerial units in providing crisis management training and to command managers to attend the proposed courses.

There was a significant relationship between previous crisis experiences and the F1 (*p* = 0.002) and F3 (*p* = 0.003) scores, which was in line with the findings of Jaafaripooyan et al. [34] but it differed from that of Wolfrum et al. [33]. Although F2 (*p* = 0.074) and F4 (*p* = 0.051) values were not significant, managers with previous crisis management experiences scored higher in these two domains as well. Based on Bandura’s theory of self-efficacy, successful experience in crisis management [46] and participation in crisis management contribute to an increase in managers’ self-efficacy. Due to the effect of crisis management experiences on F1 and F3 scores, and similar impacts of training courses on improving the F1, F2, and F4 scores, simulated crisis situations should be arranged for managers in order to raise their perceived self-efficacy through training and letting them take part in practice in full-fledged maneuvers. Participating in training courses in disaster management will help the participants become familiar with role specifications in the crisis. Accordingly, they will respond to the crisis with appropriate preparations to reduce the effects of the disaster without being perplexed [42]. Also, under the effect of performance accomplishment as a resource for improving self-efficacy [46], reviewing the lessons from previous crisis management experience seem to facilitate achieving improved self-efficacy.

The staff recruited in the incidents commandment of a medical university who are employed under a temporary contract scored lower in all four factors (F1, F2, F3, F4), although the difference was significant (*P* = 0.001); the reasons can be explained as follow: being undervalued by senior managers [47], the uncertainty of their occupational promotion and perspective, and being less included in crisis management training. In addition, temporary contract employees face a social stigma for their unstable occupational positions which leads them to inefficacy; therefore, they develop a lower perceived self-efficacy in expressing themselves and even avoid expressing constructive views which can otherwise assist organizational development and promotion [48].

In linear regression analysis, F1 (*p* = 0.003) and F2 (*p* = 0.009) scores significantly increased with the increase in participants’ years of employment (Table 5). A one-year increase in their employment history added 0.29 and 0.26 to the F1 and F3 values, respectively; this is, however, not consistent with the findings of Jaafaripooyan et al. [31].

In linear regression analysis, the manager's self-efficacy score increased significantly in the other three areas as the F2 score increased. Individuals with higher self-efficacy do not consider difficult tasks to be a threat. Rather, they look at the problem in a challenging way and try to solve it. They are committed to achieving predetermined challenging goals by planning and predicting the actions they need to take. If they fail to achieve the set goals, they will attribute failure to incomplete knowledge or inadequate effort [25]. A manager’s self-efficacy determines their ability to plan and develop preparations for responding to events and disasters.

With the increase in F3 scores, manager self-efficacy significantly increased in both F1 and F2. Individuals with higher self-efficacy exhibit superior capabilities to manage and control the situation when faced with threats and stressors [25]. On behalf of their organization, managers are responsible for coordinating and improving organizational efficacy. Achieving organizational goals depends on the characteristics and competencies of a manager, including his/her self-efficacy [23, 49].

As the F1 scores increased, so did the manager's self-efficacy in both F2 and F3. In a study by Yarmohammadian et al., commandment duties in domains defined as “*ability to notify colleagues in the event of a crisis*” and “*ability to communicate and respond in the event of a crisis*” were given higher scores than others [21], which is in line with the present findings. The areas of “*ability to find a solution to reduce staff stress*” and “*ability to attract human and financial support*” were given the lowest scores, which is not consistent with the present findings. In our present study, we categorized these domains as control, coordination, and emotional management tasks. It can be said that the ICS managers had effective commandment capabilities, and had taken measures to seek human and financial support before the occurrence of the crisis. The higher the F4 scores, the higher the F2 scores. If team members are properly oriented toward how they should function in a crisis, they will perceive fewer obstacles to their goals and the provision of professional services in the event of a crisis; they will gain more preparation to respond to the crisis as well [42]. In addition, healthcare providers will act more effectively in planning [50]; this is in line with the results of the present study.

It seems that better preparation and commandment of the pre-hospital and emergency medical services can be achieved by the promotion of its organizational level. Also, it seems essential to consider crisis managers’ abilities and attendance in relevant courses when appointing ICS managers. In designing and presentation of training; novel and creative educational methods are to be tailored to participants’ level of education and organizational position. It should be emphasized here that even training the temporary staff is an organizational investment that can return benefits to the system. This enhances their perceived self-efficacy and promotes their commitment to the organization [47]. Therefore, empowering these managers should be a priority.

## Data Availability

The corresponding author may provide you with the data sets created during this investigation.

## Abbreviations

ICS: Incidence Command System
EOC: Emergency Operations Center
WHO: Word Health Organization
KMO: Kaiser–Meyer–Olkin
